# Digitizing abdominal palpation with a pressure measurement and positioning device

**DOI:** 10.7717/peerj.10511

**Published:** 2020-12-17

**Authors:** Jia-Lien Hsu, Chia-Hui Lee, Chung-Ho Hsieh

**Affiliations:** 1 Department of Computer Science and Information Engineering, Fu Jen Catholic University, New Taipei City, Taiwan; 2 Department of General Surgery, Shin Kong Wu Ho-Su Memorial Hospital, Taipei, Taiwan

**Keywords:** Palpation, Force sensor, Abdominal physical examination

## Abstract

An abdominal physical examination is one of the most important tools in evaluating patients with acute abdominal pain. We focused on palpation, in which assessment is made according to the patient’s response and force feedback. Since palpation is performed manually by the examiner, the uniformity of force and location is difficult to achieve during examinations. We propose an integrated system to quantify palpation pressure and location. A force sensor continuously collects pressure data, while a camera locates the precise position of contact. The system recorded, displayed average and maximum pressure by creating a pressure/time curve for computer-aided diagnosis. Compared with previous work on pressure sensors of quantifying abdominal palpation, our proposed system is the integrated approach to measure palpation force and track the corresponding position at the same time, for further diagnosis. In addition, we only make use of a sensing device and a general web camera, rather than commercial algometry and infrared cameras used in the previous work. Based on our clinical trials, the statistics of palpation pressure values and the corresponding findings are also reported. We performed abdominal palpation with our system for twenty-three healthy participants, including fourteen males and nine females. We applied two grades of force on the abdomen (light and deep) by four-quadrant and nine-region schemes, record the value of pressure and location. In the four-quadrant scheme, the average pressures of abdominal palpation with light and deep force levels were 0.506(N) and 0.552(N), respectively. In the nine-region scheme, the average pressures were 0.496(N) and 0.577(N), respectively. Two episodes of contact dermal reaction were identified. According to our experiment statistics, there is no significant difference in the force level between the four-quadrant and nine-region scheme. Our results have the potential to be used as a reference guide while designing digital abdominal palpation devices.

## Introduction

An abdominal physical examination is an important tool to clinically observe the abdomen of a patient for signs of disease and cause of symptoms. An abdominal physical examination includes *inspection*, *auscultation*, *percussion* and *palpation* (Yu, 2014).

According to Jarvis, in the examination, “Physicians perform palpation to judge the size, location, and consistency of certain organs and to screen for an abnormal mass or tenderness.” (Jarvis, 2012, pp. 545). Ball et al. (2019) states that “Use palpation to assess the organs of the abdominal cavity and to detect muscle spasm, masses, fluid, and areas of tenderness.” and “Evaluate the abdominal organs of size, shape, mobility, and consistency.” (Ball et al., 2019, pp. 406).

Basic palpation involves manual compression of the abdomen to assess abdominal discomfort and pain. The results may suggest follow-up examinations using ultrasound, X-ray or computed tomography (CT) scan. For the purposes of examination and convenience in the description, the abdomen is commonly divided into four quadrants or nine regions in the more complicated scheme. In addition, Ball et al. note that “certain anatomic landmarks are useful in describing the location of pain, tenderness, and other findings.” (Ball et al., 2019, pp. 401).

The abdominal palpation generally consists of light palpation, deep palpation, liver palpation, spleen palpation, and kidney palpation (Swartz, 2002). Light palpation is useful in detecting tenderness, identifying muscular resistance and areas of muscular spasm or rigidity (Swartz, 2002; Ball et al., 2019). On light palpation, according to Jarvis, “Begin with light palpation. With the first four fingers close together, depress the skin about one cm. The objective here is to search for organs but to form an overall impression of the skin surface and superficial musculature. Save the examination of any identified tender areas until last” (Jarvis, 2012, pp. 545).

Deep palpation is necessary to thoroughly delineate abdominal organs; and to detect less obvious and abnormal abdominal masses (Swartz, 2002; Ball et al., 2019). On deep palpation, according to Jarvis, “Perform deep palpation using the same techniques described earlier (*c.f.*, light palpation) , but push down 5 to eight cm. With either technique, note the location, size, consistency, and mobility of any palpable and the presence of any abnormal enlargement, tenderness, or masses.” (Jarvis, 2012, pp. 546)

**Figure 1:**
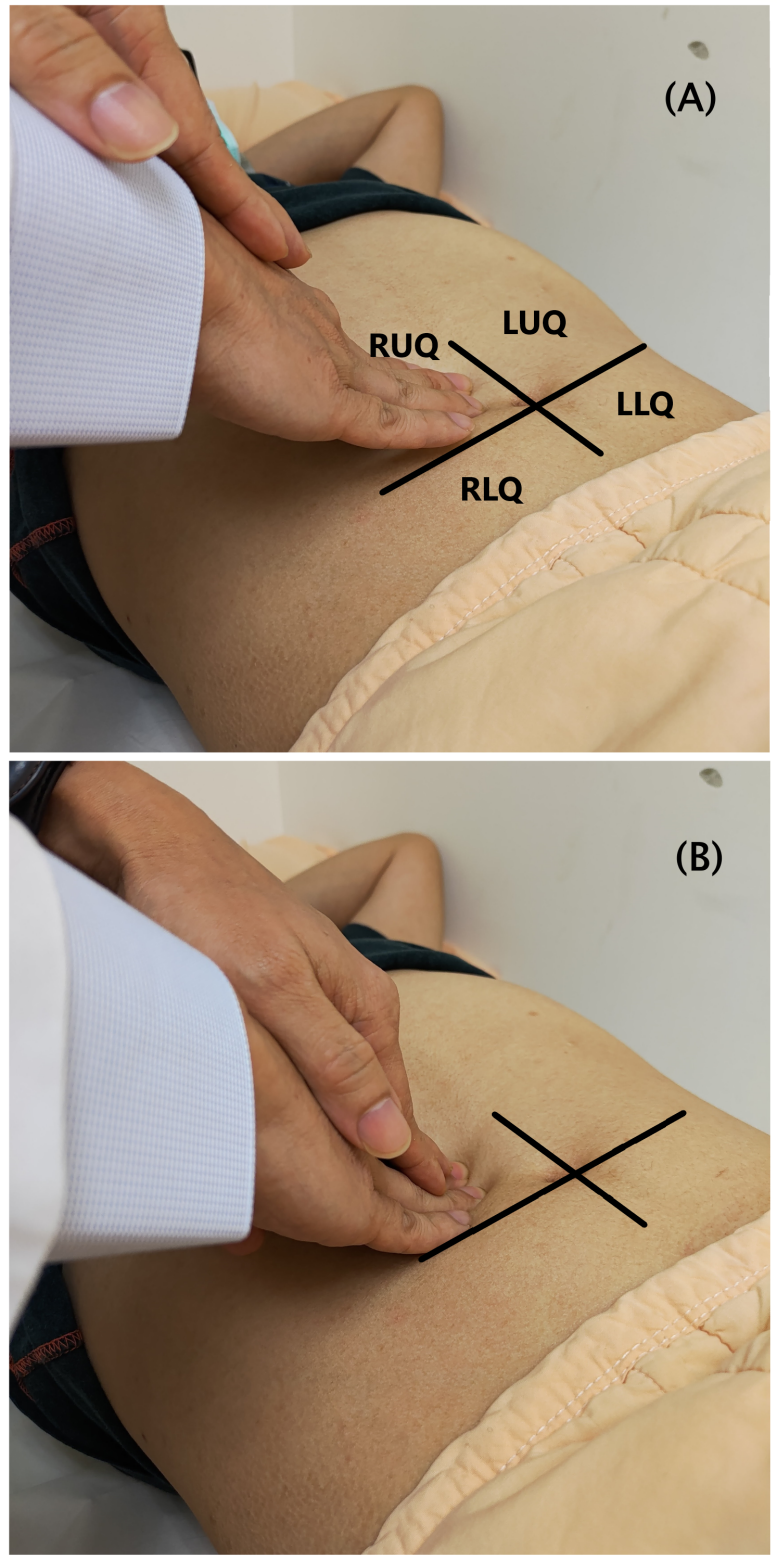
Abdominal palpation in the four-quadrant scheme. (A) Light palpation. (B) Deep palpation.

This study focuses on abdominal palpation of light palpation and deep palpation dependent on different degrees of application force. Physicians begin with light palpation by depressing the skin with three or four fingers together. Then lift the fingers and move clockwise to the next spot around the abdomen followed by deep palpation by pushing heavily towards the abdomen, as shown in Fig. 1.

Abdominal palpations are performed by physicians pressing one or both hands on the patient’s abdomen. Traditional palpation offers an advantage in terms of speed and convenience but results are imprecise. Improvements in the technology have raised the possibility of improving on the traditional palpation method through the integration of instruments or sensors during palpations or use in training simulations (Yoshitomi et al., 2016; Pacchierotti, Prattichizzo & Kuchenbecker, 2015; Tokuyasu et al., 2015).

Pressure sensors are typically classified as piezoresistive (Frantlović, Smiljanić & Lazić, 2016; Sonkar, Suja & Krishnan, 2014; Shaby & Juliet, 2011) and piezoelectric (The Institute of Electrical and Electronics Engineers, 1966; Ling, 1999; Farooq & Sazonov, 2016). Piezoresistive sensors operate on the principle that resistance values change with pressure applied to metal. The changes on the sensor indicates the changes in the pressure. Piezoelectric pressure sensors use positive and reverse piezoelectric effects. The former is a function of changes to mechanical energy, while the later reflects the conversion of electrical energy into mechanical energy. Currently, pressure-type piezoresistive sensors are more widely used due to the advantages including their lower production prices and relatively simple structure which allows them to be produced as thin sheets.

Pressure sensors have many practical applications. Currently, scan’s Flexiforce and Interlink’s Force Sensitive Resistor (FSR) sensors dominate the market. In the study (Research and Markets, 2019), the Tekscan Inc. and Interlink Electronics Inc. are leading companies. Also, in Hollinger & Wanderley (2011), the authors presented a preliminary evaluation of three commercially available force/load sensors, including the Interlink FSR, the LuSense PS3 and the Tekscan Flexiforce. The Flexiforce showed the highest precision (i.e., the quality that characterizes the capability of a measuring instrument of giving the same reading when repetitively measuring the same quantity under the same prescribed conditions). Also, the Flexiforce showed the slowest response (time to reach 90% of its final resistance value).

When comparing these two sensors, Vecchi et al. (2001) found that the Flexiforce sensors are more expensive but provides more accurate and consistent measurement with faster dynamic testing and longer offset measurement. In Vecchi et al. (2001), “The Flexiforce sensors showed better performance in terms of repeatability, linearity and time drift when mounted on a rigid substrate, and in terms of dynamic accuracy when mounted on the thumb.” Considering the static calibration, the standard deviations in percentile as regards to the full scales of 30N are 1.6% (Flexiforce) and 6.8% (FSR). During the repeatability tests, the maximum percentage errors generated by the two types of sensors are 2.2% (Flexiforce) and 5.1% (FSR). Considering the dynamic force measurement accuracy, the standard deviation of the measured error during dynamic force measurements for the Flexiforce sensor and the FSR sensor over 0-20N force range are 1.8 and 3.1. Moreover, the Flexiforce sensors showed a better dynamic accuracy than the FSR sensors. Considering the sensor cost, the purchase price of FlexiForce A201 Sensor is US$73.3 (4-pack), and the price of FSR Model 408 is US$3.99. Note that, the price is directly obtained from the company website (FlexiForce A201 Sensor, 2020; Interlink Electronics, 2020), at 2020/10/28.

Castro & Cliquet (1997) proposed a set of wearable pressure sensors and angle gauges to measure the force applied by the thumb, index and middle fingers along with the degree of elbow flexion. Tests were conducted with 30 subjects engaged in drinking water from a cup held in the hand to determine whether the method could be used as a standard for action detection. Nam, Kim & Kim (2016) designed a pressure sensor integrated into a belt with an accelerometer to detect changes in the posture between sitting and standing to determine proper sitting posture. Here, we use a pressure sensor to measure and record force applied in manual palpation.

Other studies including (Praveen & Guhilot, 2013; Cha et al., 2013; Ogris, Kreil & Lukowicz, 2007; Kang et al., 2011; Sepúlveda et al., 2011; Zhang et al., 2001) have applied pressure sensors in a range of areas to detect contact or separation of objects, as triggers or switches to activate subsequent events or to measure pressure. Such sensors can also be combined with wireless communications modules. Park et al. (2016) used multiple-sensors to detect reaction force while walking using the original pressure sensor to design a pressure detection system.

Katsuki et al. (2015) designed a sensor system for an abdomen palpation procedure in traditional medicine. The sensor system makes use of the custom-designed glove, in which the detecting circuit mounted on, to measure the depth and the force during the abdominal palpation. Katsuki combined a pressure sensor and an activity capture system for use in abdominal examinations using Bluetooth for data transmission. Palpation force and depth data are displayed in a graphical interface. The pressure sensor is installed in a glove and directly measures force during palpation. While this approach is highly intuitive, incorrect measurement values can result from the sensor inadvertently changing position during measurement. Others have tested the range of pressure sensors with Krklješ, Nagy & Babkovič (2012) using points of contact with different area measurements and finding sensors with a dome-type face to produce more accurate measurement values.

Algometer is an instrument for measuring sensitivity and intensity of pain caused by pressure. According to the studies (Ko et al., 2016; Kinser, Sands & Stone, 2009) algometer can be a reliable and valid diagnostic device for abdominal examination. It has the possibility of practical use for quantification and standardization of abdominal examination. For instance, Wieckiewicz et al. (2015) made use of algometer to determine the diagnostic value of pressure algometry in the temporomandibular disorders. Wytrazek et al. (2015) performed an algometer test for pressure pain threshold to assess trigger points and the activity of motor units in the neck and shoulder girdle muscles for their detection.

In addition to abdominal palpation, some sensoring approaches of pressure assessment have been proposed for certain symptoms, causes, and treatment in clinical trials. For example, Niederauer et al. (2020) design a vaginal dynamometer that utilizes an intra-abdominal sensor to obtain vaginal closure force for measuring pelvic health. Masuda et al. (2018) reported that palpation of the masseter muscle with three mechanical stimuli (0.5 kg, 1.0 kg, or 2.0 kg) may evoke referred pain in healthy individuals.

Myers et al. (2017) design a telehealth system which guides a patient through an exact probing motion that precisely matches the palpation motion set by the physician. Thus, the patient is able to conduct a remote palpation of the physical examination by only using a smartphone with native accelerometers. In this study, four kinds of palpation motion (i.e., deep+slow; shallow+slow; deep+fast; shallow+fast) are correlated with the single-axis acceleration quantities.

However, the assessment process of palpation is susceptible to considerable variation. Applied force levels might differ between examiners and even the same examiner might apply different levels of force. Light and deep palpations are only defined in the descriptive manner and by the approximate range of depress depth (Swartz, 2002; Ball et al., 2019; Jarvis, 2012). In addition, patients may differ in terms of their sense of tenderness and tolerance.

Further, we make use of the sensing rod as a measuring medium in which the pressure value is obtained by the pressure sensor. In our design, we choose the solid sensing rod rather than “pressure-sensing glove”, since the glove may not perfectly fit the examiner’s hand. In addition, the sensor of glove is usually at the fingertip of the glove and it may result in shift and misplace. When performing the palpation, the applying pressure at the fingertip must be very firm and secure. Otherwise, the reading from the fingertip sensor may not be stable during use. As a result, the uniformity of force and location is difficult to achieve during examinations. However, the use of the sensing rod ensures that readings obtained under normal operation are not affected by these factors.

This purpose of this study is to design a system for quantifying and recording palpation pressure by numeric values and using a video camera to automatically detect palpation positions. The pressure value and corresponding position of each palpation is assessed and a series of palpation data over time is presented and visualized as a graph for clinical decision support.

## Materials & Methods

### System architecture

**Figure 2:**
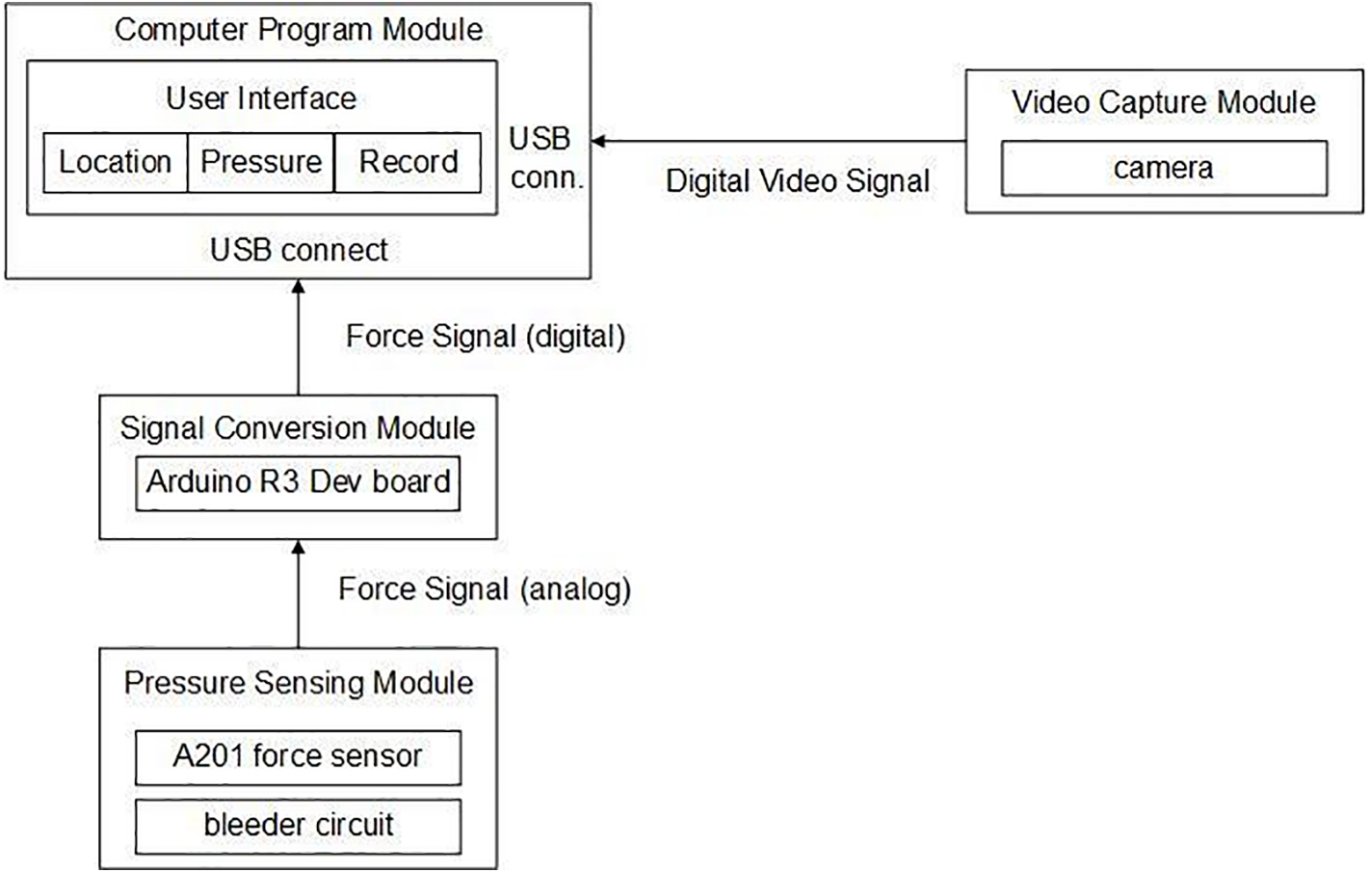
System architecture.

In reference to Fig. 2, the system architecture consists of four modules: computer program, pressure sensor, signal conversion and video capture. The pressure sensing module uses a pressure sensor to produce a palpation pressure value and then a voltage divider circuit is used to transfer the potential signal to the signal conversion module. The signal conversion module is implemented using the Arduino R3 development board to digitize the analogue signal produced by the pressure sensing module. The video signal from the camera is simultaneously sent to the computer program module to assess the position of sensing rod and determine the corresponding locations in the four-quadrant or in the nine-region scheme. This digitized data is processed and presented in the user interface as a real-time monitoring image and the corresponding pressure/time curve.

### Single chip processor combined with pressure measurement and positioning

**Figure 3:**
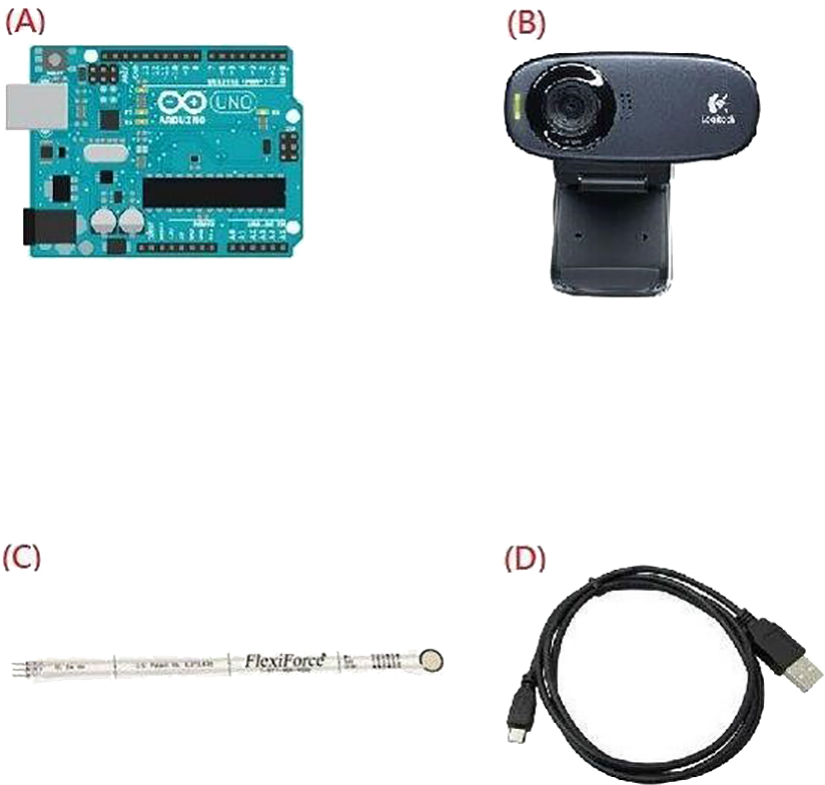
Four components of the proposed system. (A) Arduino R3 development board, (B) Logitech C310 video camera, (C) A201 pressure sensor, (D) USB cable.

The proposed pressure and positioning measurement system uses the following components (as shown in Fig. 3):

(A) Arduino R3 development board.

(B) Logitech C310 video camera.

(C) Flexiforce A201 pressure sensor.

(D) USB cable.

(A) Arduino R3 development board, (B) Logitech C310 video camera, (C) A201 pressure sensor, (D) USB cable.

**Figure 4:**
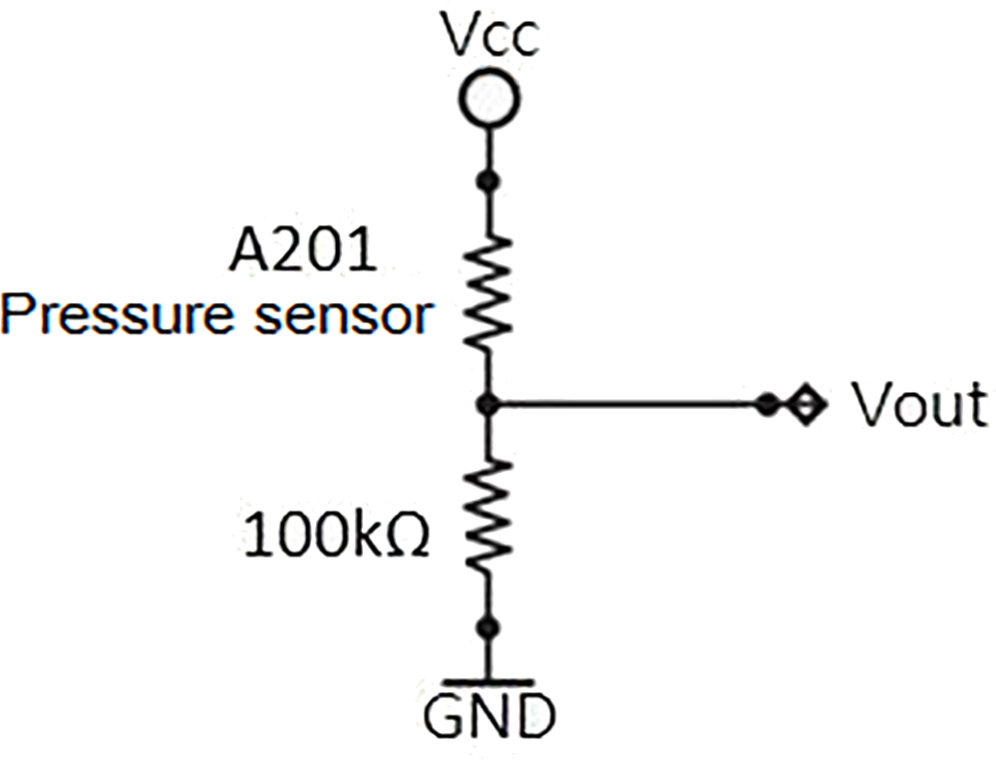
Dividing circuit: The A201 pressure sensor and a fixed resistor in series, using the Vcc side with an input voltage of 5V, the Vout can be measured by the pressure sensor sub-voltage.

In our system, we chose a Tekscan Flexiforce A201 piezoresistive pressure sensor. The sensor is a thin plate that can detect the pressure value at the desired location. As pressure increases, the resistance value shrinks. The pressure value is determined directly by measuring the sensor’s internal resistance or by measuring the voltage across the sub-voltage circuit to determine the relative pressure value (Fig. 4).

In a series voltage circuit, the voltage is inversely proportional to the resistance, thus the measured voltage value can be used to determine the resistance. Once the sub-voltage is read, the Arduino R3 development board is used for signal conversion. The Arduino R3 development board is an open-source development platform that uses the ATmega328P as its main processor with digital/analogue input and output terminals to control electronic components and integrate sensors.

It is suitable for the development of various types of sensor modules or Internet of Things applications and it is used here for signal conversion to reduce computational requirements and ensure smooth operations. In our system, the sampling rate of data acquisition is 20 Hz.

**Figure 5:**
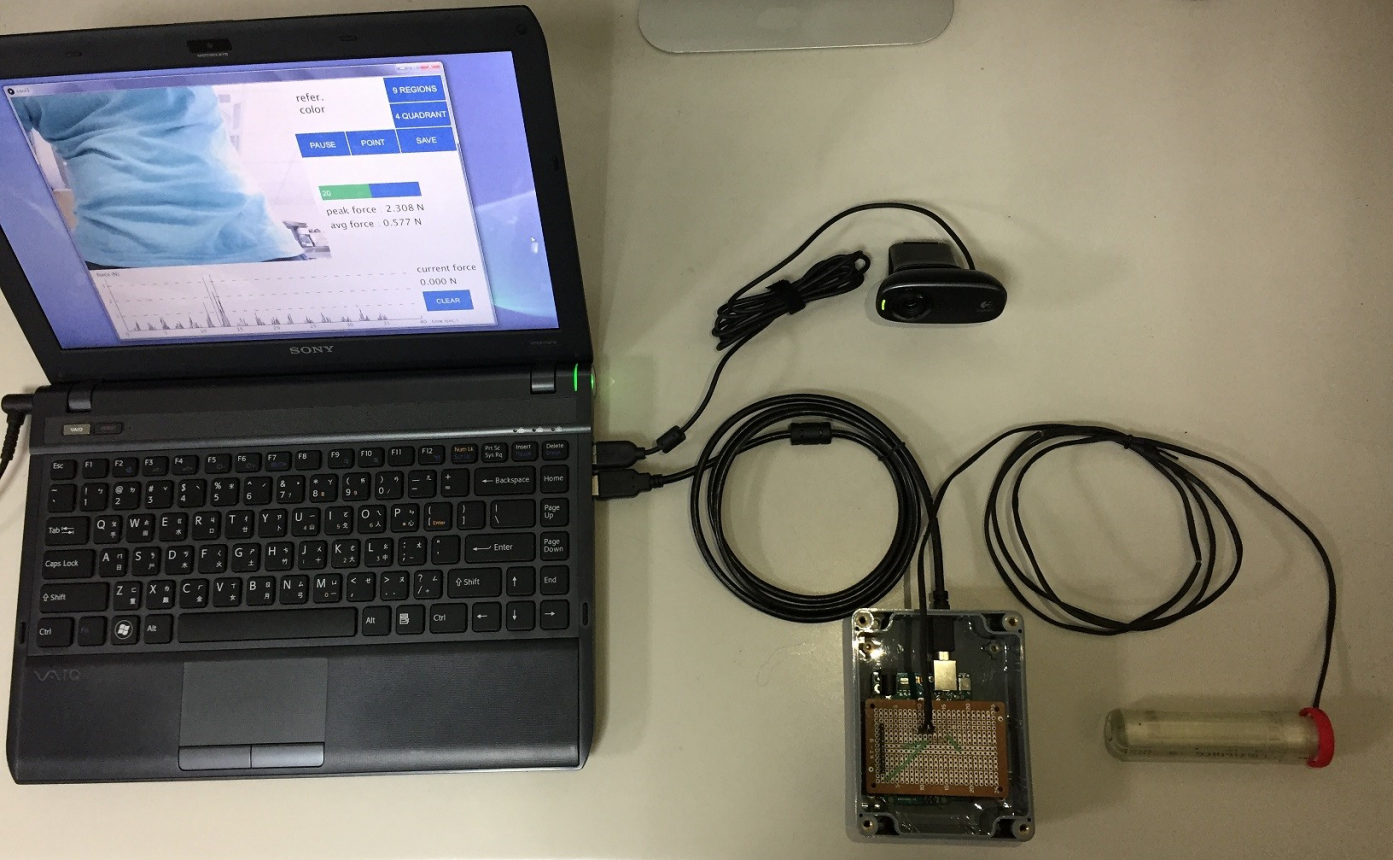
The connected environment with the force-sensing and location positioning device.

**Figure 6:**
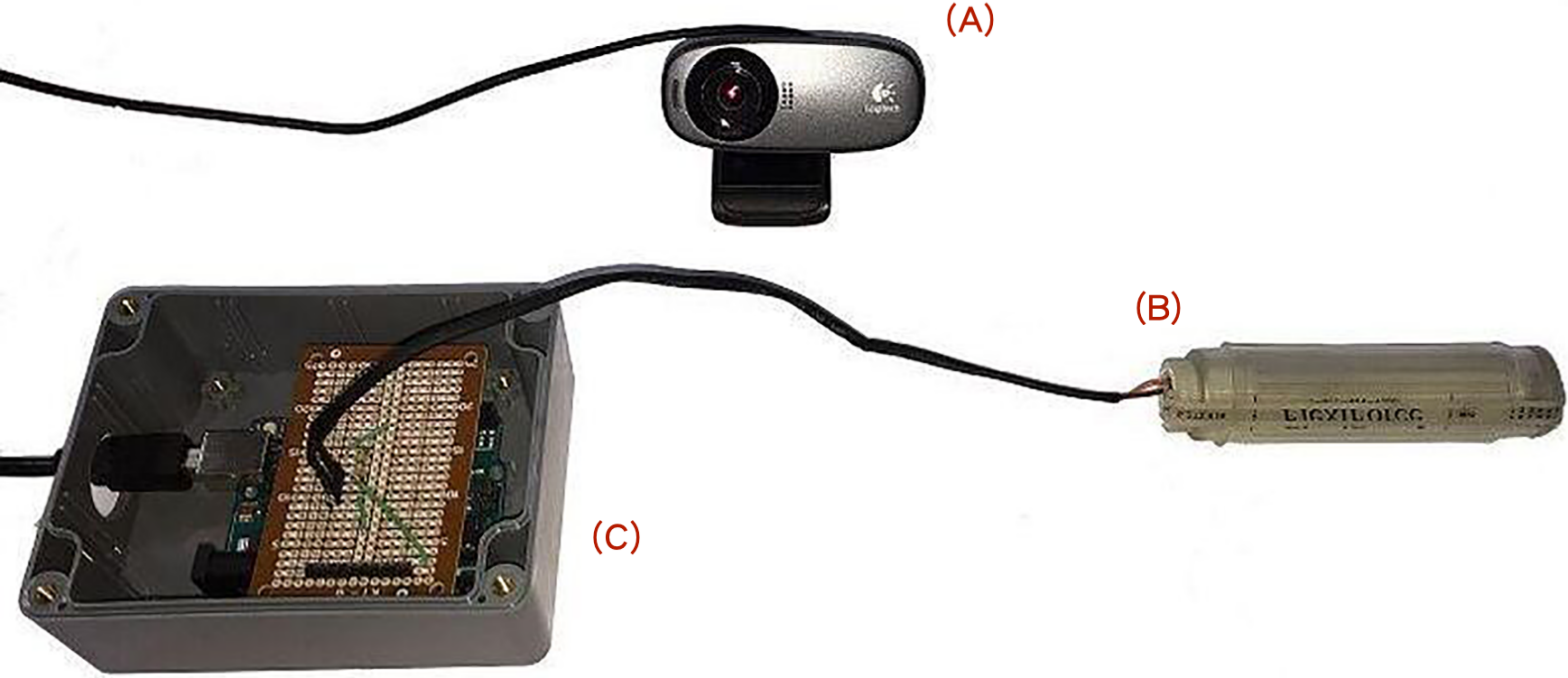
Component connection diagram: Video camera (A), sensing rod (B), box containing the divided circuit and the Arduino R3 development board (C).

These components are connected through the computer (Fig. 5) and components are shown in Fig. 6. The pressure sensor is positioned at the end of the sensing rod. We then connect the sub-voltage circuit and the Arduino R3 development board and these are packaged to prevent accidental damage. The Arduino R3 development board and the video camera are connected to the computer via the USB cable. The computer software integrates all the data and provides a user interface for operation.

**Figure 7:**
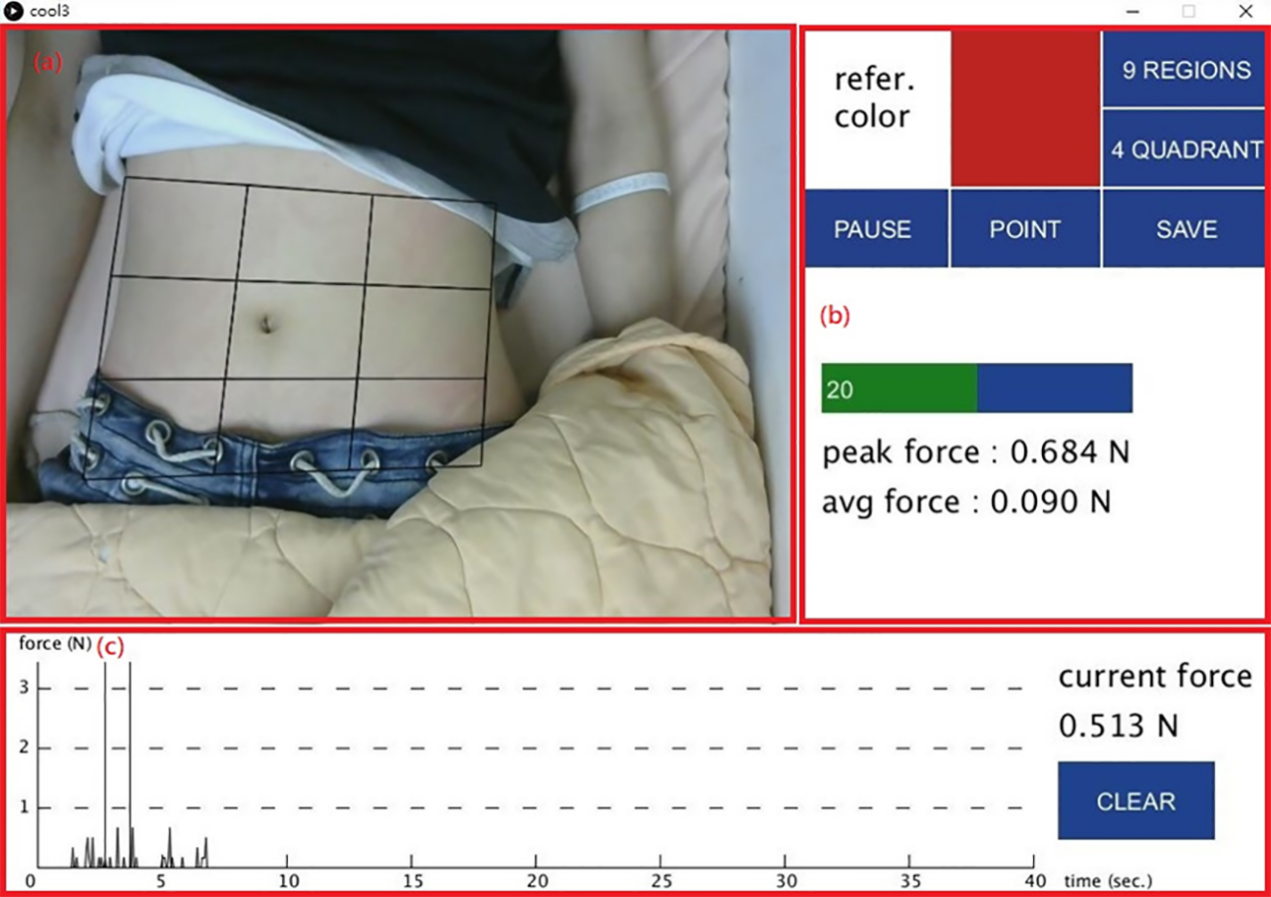
The user interface. (A) A grid is superimposed over a live video. (B) Control button. (C) A graph shows palpation pressure over time.

In addition, we design a location-positioning mechanism while performing abdominal examinations. A video screen camera provides a real-time visual along with a positioning marker for the initial palpation. Note that the marker is the color of the end of the sensing rod. In reference to Fig. 6, the color of marker is red which matches the user-specified “refer.color” (in Fig. 7). The proposed system keeps tracking the sensing rod and obtains continuous measurements from the current sensing rod on the detection area.

### Software design and user interface

All the four components are integrated into a core system by using two development kits, Arduino IDE and processing. Arduino IDE allows the user to control sensor reading, electronic components and monitor sensor data. The processing is a flexible software package that provides a user-friendly development environment for a quick application interface. These two software packages are compatible and we use the Arduino IDE to read sensor signals for conversion by the Arduino R3 development board. The processing is then used to design a user interface to present all data signals.

Figure 7 shows the user interface: (a) a grid is superimposed over a live video feed of the subject’s abdomen; (b) control buttons of which “refer.color” allows the user to select a reference color for palpation points on the screen for tracking, “PAUSE” temporarily halts sensor reading, “POINT” stores the pressure value of a single palpation, “SAVE” outputs all test results to the log file with the peak and average force values immediately displayed below; (c) a graph that shows palpation pressure over time with current pressure value displayed on the right. The “CLEAR” button zeros out current pressure values.

### Experiments

Experiments were carried out on twenty-three subjects to collect palpation data in terms of pressure distribution in different areas. Experimental results show the abdominal examination area and corresponding pressure value to provide quick diagnostic information, which will be detailed in ‘Results’.

### Ethics statement

The study was approved by the Institutional Review Board of Shin Kong Wu Ho-Su Memorial Hospital, Taiwan, R.O.C. The IRB number was 20160808R. Written informed consents were obtained from all participants.

### Experimental setup: procedure and steps

**Figure 8:**
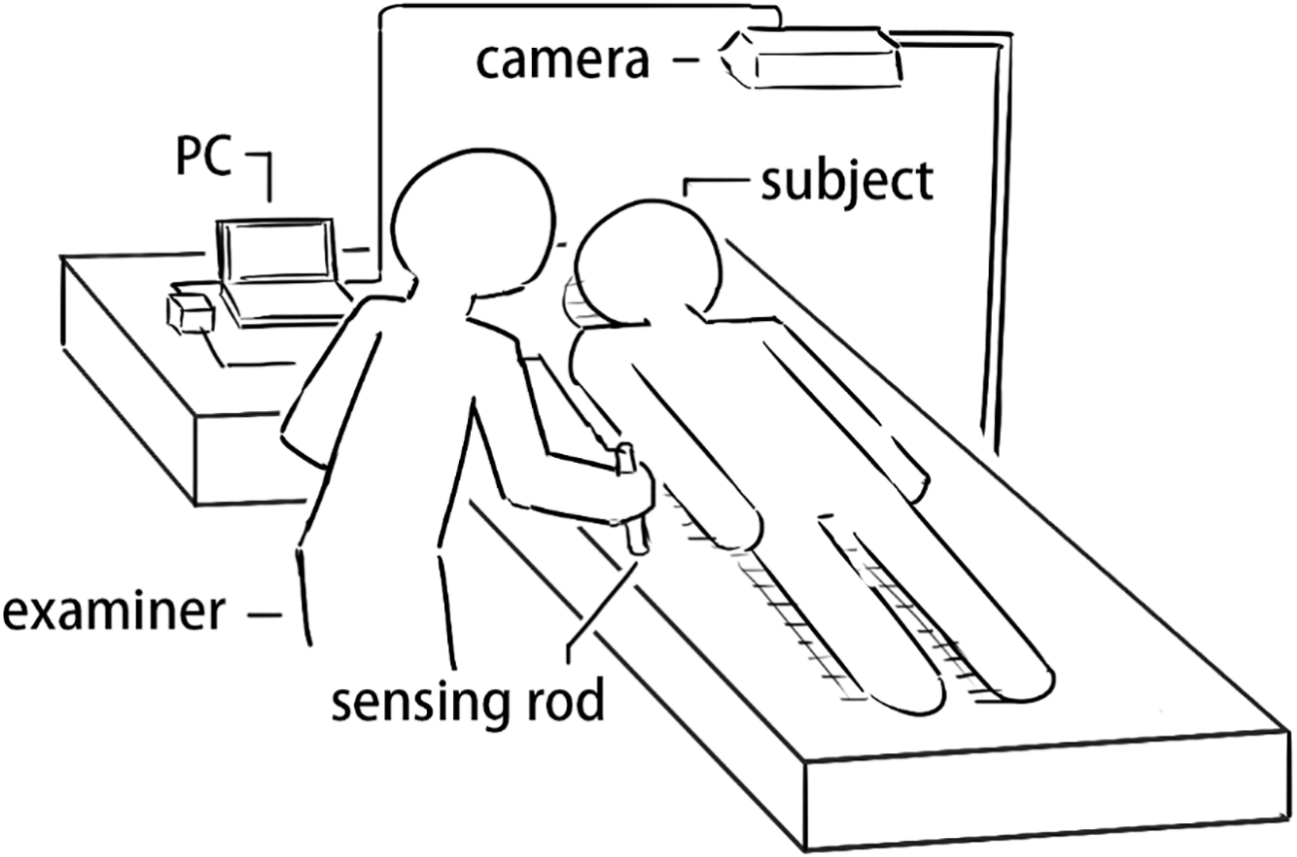
Experimental setup.

Abdominal palpations were performed by a surgeon with experience more than twenty years on twenty-three subjects at clinical skill center of Shin Kong Wu Ho-Su Memorial Hospital. Figure 8 shows the experimental procedure in which the sensing rod is used for palpation with the camera used to detect the palpation location. This data is then processed and displayed by the computer for computer-aided diagnosis in the user interface.

### Experimental procedure and steps

The experimental process starts with an abdominal palpation examination, beginning with light palpations followed by deep palpations to prevent deep palpations from affecting the results of subsequent light palpations in the four-quadrant scheme and nine-region scheme, respectively.

1. The subject lies supine with the abdomen exposed.
2. At system initialization, the examiner selects a reference color in the interface to mark the sensing rod for tracking.
3. The examiner locates the umbilicus and the four corners of the sensing area includes the points of anterior superior iliac spines and the cross of anterior axillary line and costal margin and using the sensing rod for trapezoidal correction to overlay a grid over the abdomen.
4. The system shows the current, maximum and average pressure values in a pressure/time curve.
5. After the examiner presses the sensor rod at the certain area and clicks the POINT button to record a single pressure measurement. The PAUSE button then pauses the measurement. The SAVE button exports all measurements to storage as a text file while CLEAR zeros out the current values.

**Figure 9:**
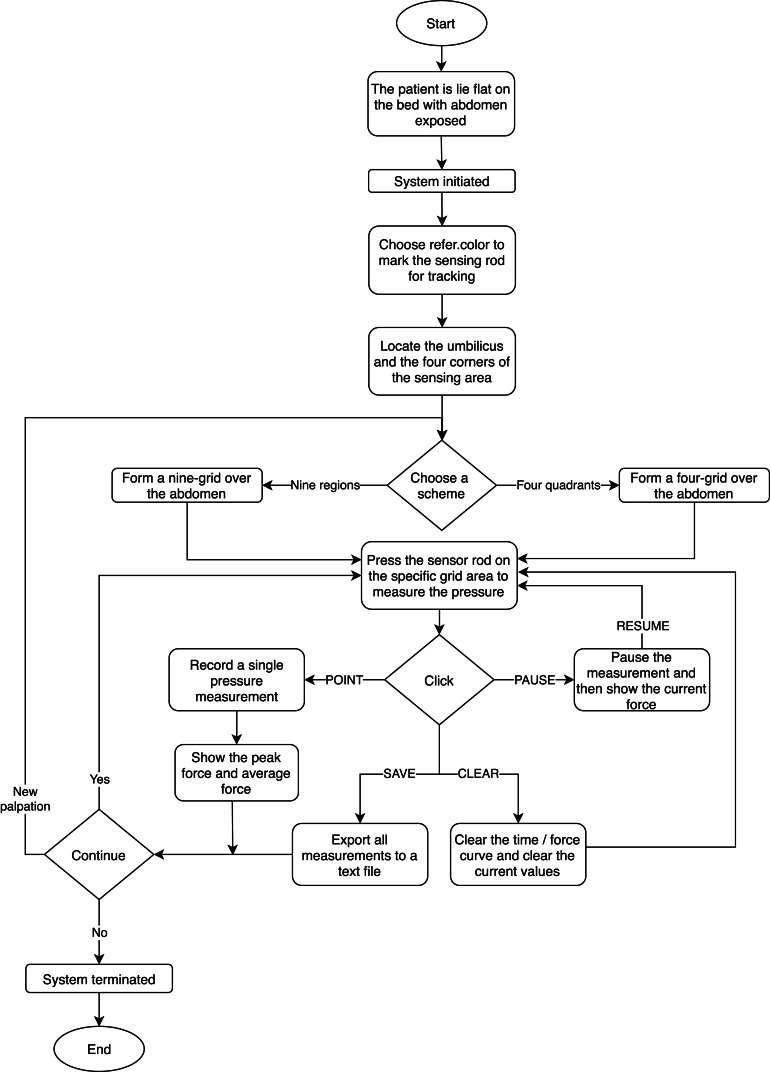
The flowchart of the experimental process.

Note that, each palpation in an area will be performed repeatedly (usually, three or four times). In addition, we illustrate the experimental process in the flowchart, as shown in Fig. 9.

## Results

We performed abdominal palpation with our system for twenty-three healthy participants including fourteen males and nine females with a mean age of 32 years (range 23–49). During all the examination and before, participants did not report any pain. One thousand four hundred and forty-six light palpations and one thousand four hundred and forty-four deep palpations were performed. Two episodes of contact dermal reactions were found and was released spontaneously after one hour’s observation.

In reference to Tables 1, 2, 3, and 4, we present the statistics of pressure values of light and deep palpation in the four-quadrant and nine-region scheme, respectively.

**Figure 10:**
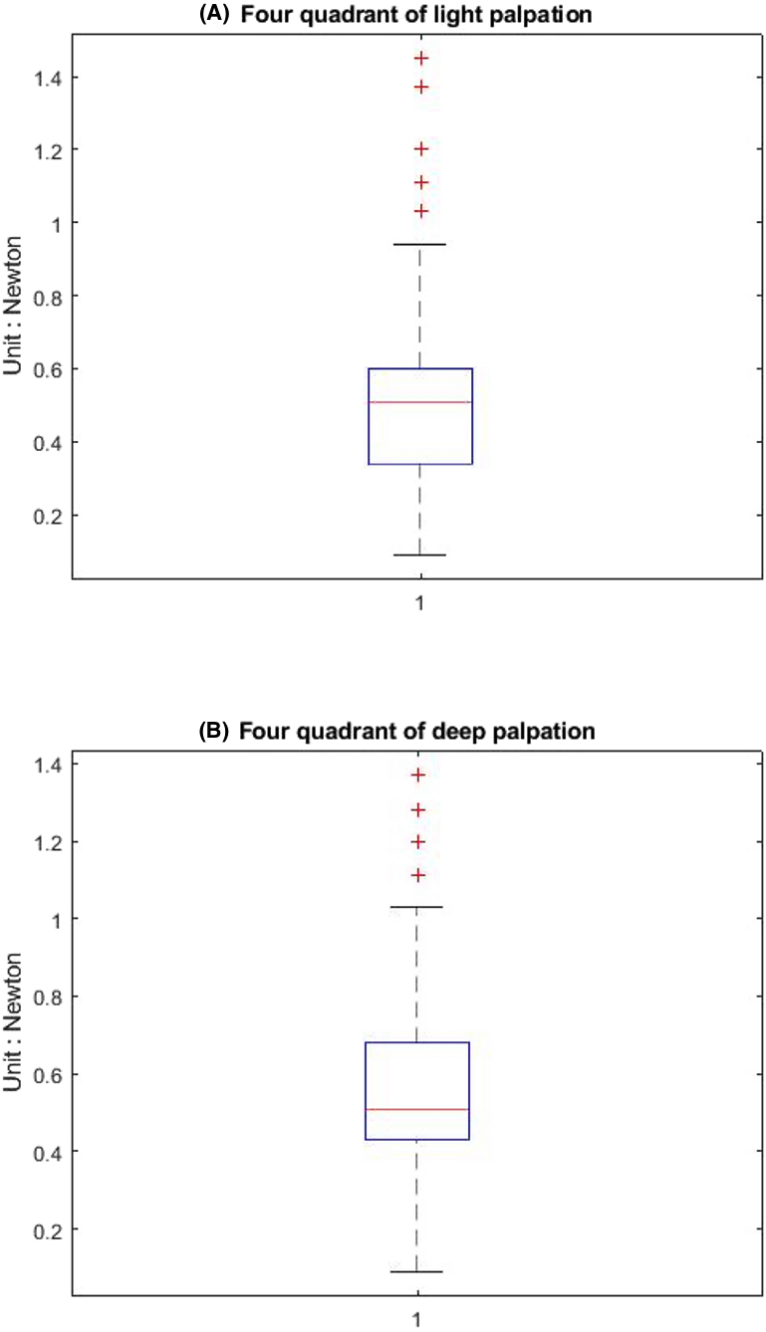
The box-plot of four-quadrant palpation pressure. (A) Light palpation pressure in the four-quadrant scheme. (B) Deep palpation pressure in the four-quadrant scheme.

**Figure 11:**
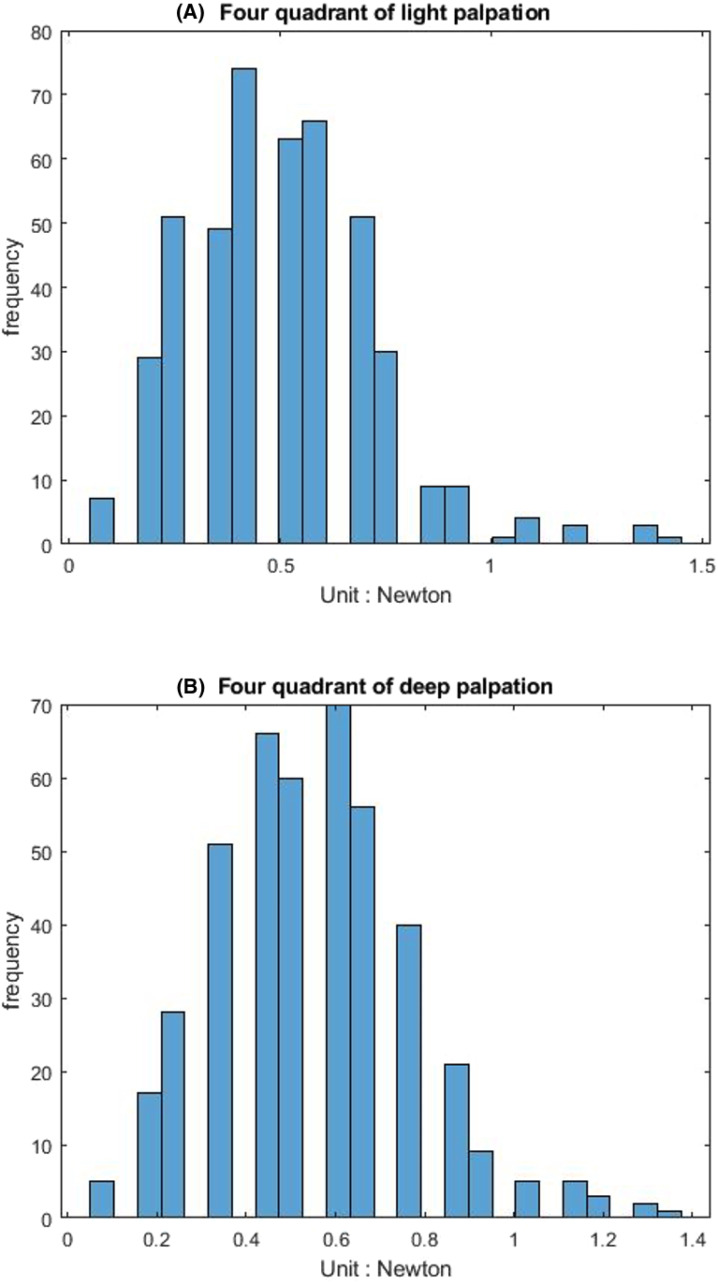
The histogram and distribution of four-quadrant palpation pressure. (A) Light palpation pressure in the four-quadrant scheme. (B) Deep palpation pressure in the four-quadrant scheme.

**Figure 12:**
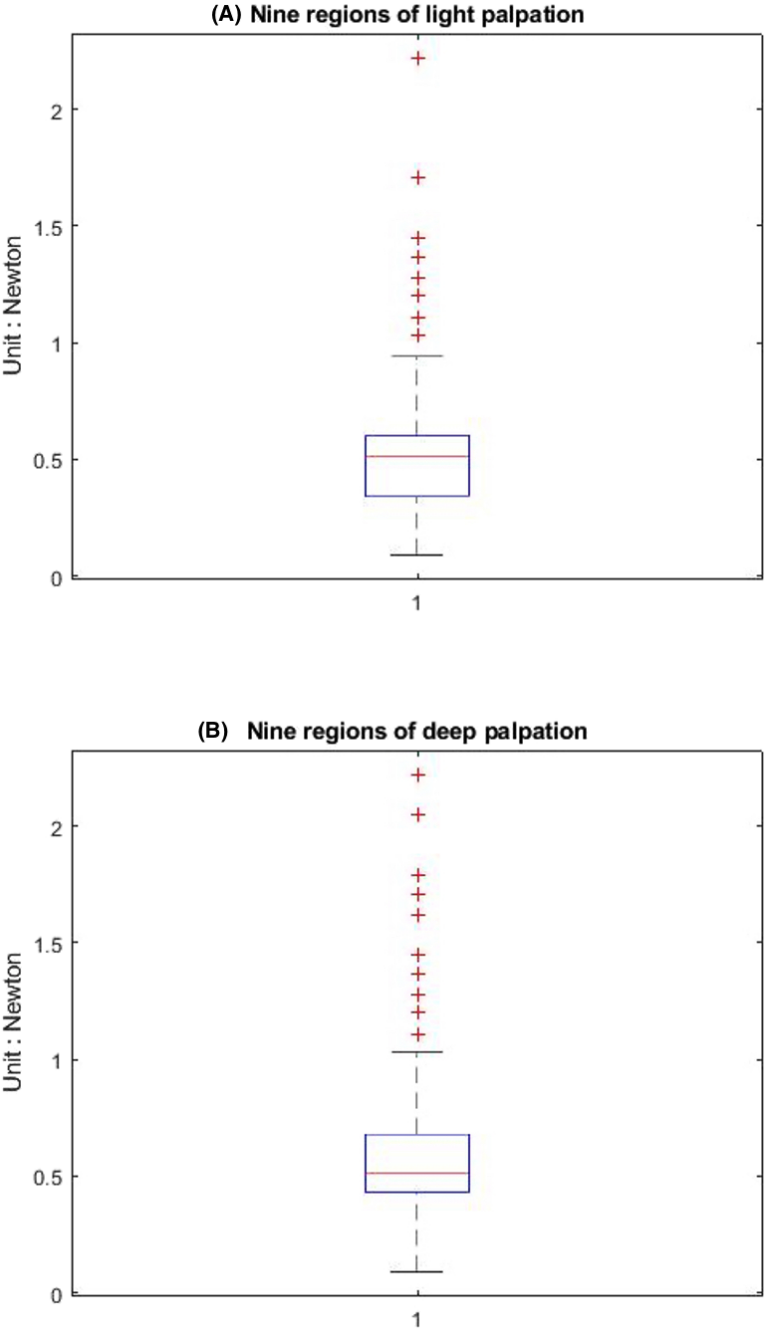
The box-plot of nine-region palpation pressure. (A) Light palpation pressure in the nine-region scheme. (B) Deep palpation pressure in the nine-region scheme.

**Figure 13:**
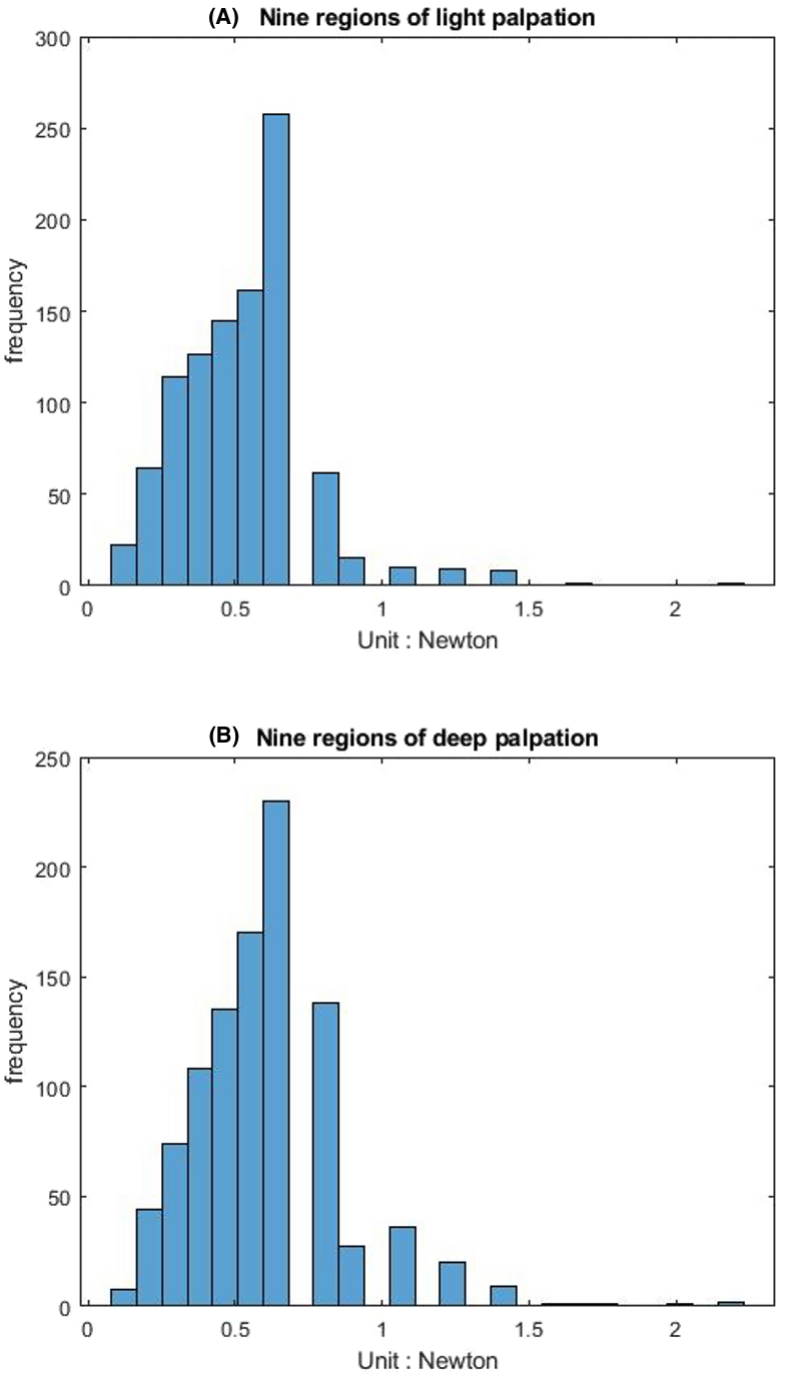
The histogram and distribution of nine-region palpation pressure. (A) Light palpation pressure in the nine-region scheme. (B) Deep palpation pressure in the nine-region scheme.

Table 1 shows that the light palpation force exerted in the four quadrants ranges between 0.485N and 0.527N, with an average force of 0.506N, while Table 2 shows that for deep palpations ranged between 0.532N and 0.573N, with an average force of 0.552N. Table 3 shows that the nine regions light palpation force ranged between 0.481N and 0.510N, with an average of 0.496N, while Table 4 shows that for deep palpations ranged from 0.560N to 0.594N, with an average of 0.577N. For better illustration, we show the box plot and histogram and distribution of the palpation pressure in Figs. 10, 11, 12 and 13.

## Discussion

Pressure sensors have been used in the evaluation of local tenderness with algometry (Fenton et al., 2009). Some studies used pressure sensors to quantify abdominal palpations (Katsuki et al., 2015). In the work (Fenton et al., 2009), the authors focus on the chronic pelvic pain (CPP) to perform the study of pain pressure threshold (PPT) of the lower anterior abdominal wall in CPP patients; and to determine the range and distribution of values at every 14 site, and the clinical utility of using PPT in a definition of termed myofascial pain syndrome (MFPS). However, our proposed system is an integrated approach to continuously keep track the palpation force and location information for further diagnosis. In the work (Katsuki et al., 2015), the authors design the system, which makes use of a motion capture system and a thin film force sensor mounted at a glove, for an abdominal palpation procedure to measure the depth and the force/pressure at the same time during abdominal palpations. However, in the system, the plural infrared cameras and reflective markers are required to setup the testing environment. On the contrary, in our system, we only need a general web camera, and the force sensor attached to a solid rod.

In our study, we combined abdominal palpation pressure values with visual and positioning information, which may have the potential to be more helpful in identifying the cause of abdominal pain. Experimental testing provides a range of clinical pressure measurements for light and deep palpations allowing multiple subsequent operators to differentiate between them. Then, we investigate differences of light palpation pressure between the four-quadrant and nine-region scheme, as well as the differences between light and deep palpation.

**Table 1:** The pressure value of light palpation in the four-quadrant scheme. 95% Confidence interval. (0.485, 0.527).

**Region**	**Palpation counts**	**Mean ±SD (unit: N)**	**Max.**	**Min.**
RUQ	112	0.535 ± 0.241	1.37	0.09
LUQ	111	0.482 ± 0.198	1.11	0.09
RLU	114	0.499 ± 0.214	1.37	0.09
LLQ	113	0.507 ± 0.241	1.45	0.09
All quadrants	450	0.506 ± 0.225	1.45	0.09

**Notes.**

RUQ right upper quadrant LUQ left upper quadrant RLQ right lower quadrant LLQ left lower quadrant

**Table 2:** The pressure value of deep palpation in the four-quadrant scheme. 95% Confidence interval (0.532, 0.573).

**Region**	**Palpation counts**	**Mean ±SD (unit: N)**	**Max.**	**Min.**
RUQ	110	0.568 ± 0.226	1.28	0.09
LUQ	108	0.560 ± 0.219	1.11	0.17
RLU	108	0.544 ± 0.222	1.37	0.09
LLQ	113	0.537 ± 0.215	1.28	0.09
All quadrants	439	0.552 ± 0.221	1.37	0.09

**Notes.**

RUQ right upper quadrant LUQ left upper quadrant RLQ right lower quadrant LLQ left lower quadrant

**Table 3:** The pressure value of light palpation in the nine-region scheme. 95% Confidence interval (0.481, 0.510).

**Region**	**Palpation counts**	**Mean ±SD (unit: N)**	**Max.**	**Min.**
Right Hypochondriac	111	0.495 ± 0.204	1.28	0.09
Epigastric	111	0.521 ± 0.271	2.22	0.09
Left Hypochondriac	113	0.534 ± 0.248	1.71	0.17
Right Lumbar	110	0.476 ± 0.236	1.45	0.09
Umbilical	107	0.506 ± 0.244	1.37	0.09
Left Lumbar	111	0.512 ± 0.231	1.37	0.09
Right Inguinal	112	0.459 ± 0.225	1.28	0.09
Hypogastric	113	0.474 ± 0.198	1.03	0.09
Left Inguinal	108	0.483 ± 0.218	1.37	0.09
All regions	996	0.496 ± 0.232	2.22	0.09

**Table 4:** The pressure value of deep palpation in the nine-region scheme. 95% Confidence interval (0.560, 0.594).

**Region**	**Palpation counts**	**Mean ±SD (unit: N)**	**Max.**	**Min.**
Right Hypochondriac	111	0.579 ± 0.234	1.28	0.09
Epigastric	105	0.582 ± 0.239	1.45	0.17
Left Hypochondriac	115	0.537 ± 0.225	1.28	0.17
Right Lumbar	111	0.560 ± 0.250	1.28	0.09
Umbilical	112	0.666 ± 0.346	2.22	0.09
Left Lumbar	110	0.590 ± 0.346	2.22	0.09
Right Inguinal	116	0.544 ± 0.252	1.45	0.09
Hypogastric	114	0.561 ± 0.251	1.45	0.09
Left Inguinal	111	0.573 ± 0.257	1.45	0.17
All regions	1005	0.577 ± 0.272	2.22	0.09

First, we apply the Wilcoxon rank-sum test analysis to independent palpation samples. The Wilcoxon rank-sum test (also called the Mann–Whitney U test) is a nonparametric test of testing whether two samples are likely to derive from the same population.

In this study, we apply the ranksum function of Matlab returns the *p*-value of a two-sided Wilcoxon rank sum test. The ranksum tests the null hypothesis that data in *x* and *y* are samples from continuous distributions with equal medians, against the alternative that they are not.

For the both groups (four-quadrant and nine-region) during light palpations, *p*-value = 0.3060 indicates that there is not enough evidence to reject the null hypothesis at the default significance level 5%. That is, no significant difference was found between the two schemes for light palpations.

During deep palpations, *p*-value = 0.5346 indicates that there is not enough evidence to reject the null hypothesis at the default significance level 5%. That is, no significant difference was found between the two schemes for deep palpations.

**Figure 14:**
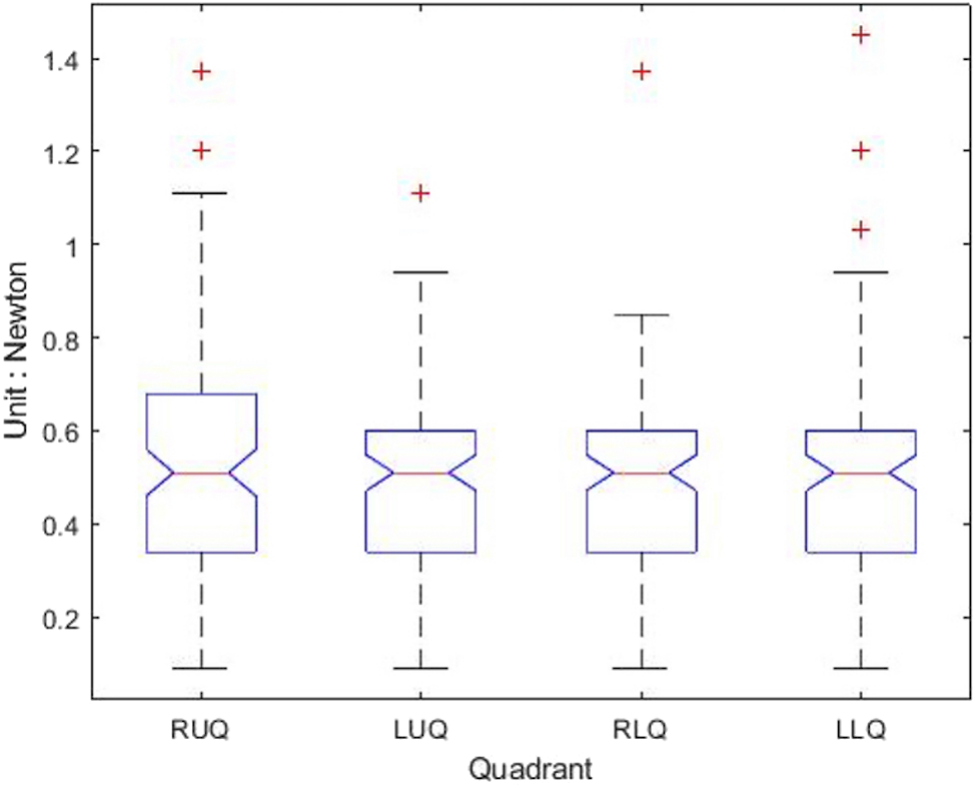
The box-plot of four-quadrant light palpation for each group.

Moreover, when performing light palpation in the four-quadrant scheme, all palpations in the same quadrant are considered a group. That is, we have four groups. We apply the Kruskal-Wallis test analysis to independent palpation groups. The Kruskal-Wallis test is a nonparametric method for testing whether samples originate from the same distribution. Table 5 shows the output and Fig. 14 shows the box-plot of the Kruskal-Wallis test and whether there is a statistically significant difference between the four groups of light palpation. We can see that the *p*-value = 0.5057, which is above 0.05. Therefore, there is no statistically significant difference of light palpation performed in each quadrant.

**Figure 15:**
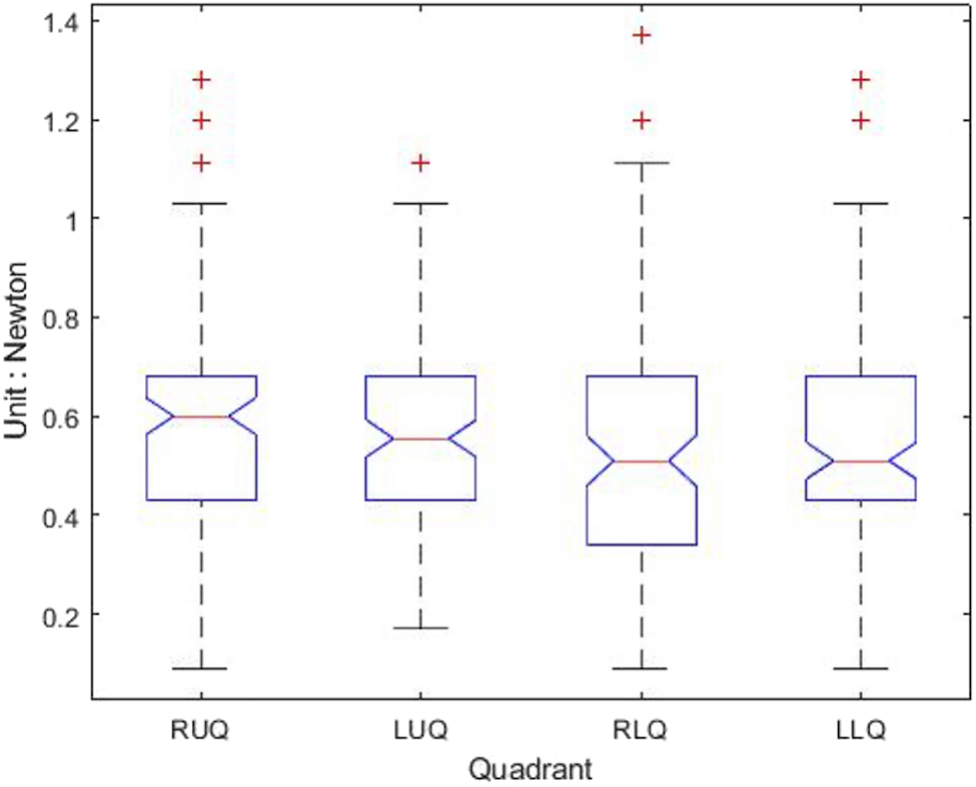
The box-plot of four-quadrant deep palpation for each group.

Similarly, Table 6 shows the output and Fig. 15 shows the box-plot of the Kruskal-Wallis test and whether there is a statistically significant difference between the four groups of deep palpation. We can see that the *p*-value = 0.8018, which is above 0.05. Therefore, there is no statistically significant difference of deep palpation performed in each quadrant.

**Figure 16:**
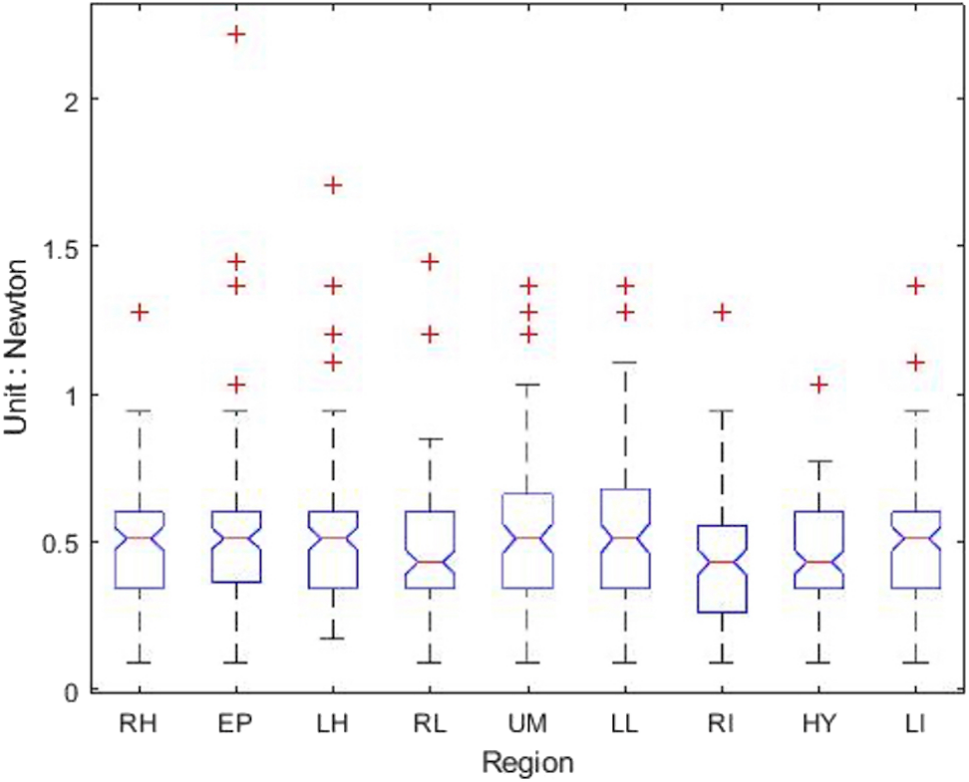
The box-plot of nine-region light palpation for each group.

Considering the nine-region scheme, Table 7 shows the output and Fig. 16 shows the box-plot of the Kruskal-Wallis test and whether there is a statistically significant difference between the nine groups of light palpation. We can see that the *p*-value = 0.2089, which is above 0.05. Therefore, there is no statistically significant difference of light palpation performed in each region.

**Table 5:** The Kruskal–Wallis ANOVA Table of light palpation in the four-quadrant scheme (variable: palpation pressure; groups: four quadrants).

Source	SS	df	MS	Chi-sq	Prob>Chi-sq
Groups	38906.2	3	12968.7	2.34	0.5057
Error	7439924.8	446	16681.4		
Total	7478831	449			

**Table 6:** The Kruskal-Wallis ANOVA Table of deep palpation in the four-quadrant scheme (variable: palpation pressure; groups: four quadrants).

Source	SS	df	MS	Chi-sq	Prob>Chi-sq
Groups	15820.02	3	5273.3	1	0.8018
Error	6929887.48	435	15930.8		
Total	6945707.5	438			

**Table 7:** The Kruskal–Wallis ANOVA Table of light palpation in the nine-region scheme (variable: palpation pressure; groups: nine regions).

Source	SS	df	MS	Chi-sq	Prob>Chi-sq
Groups	885535.6	8	110692	10.87	0.2089
Error	80143660.9	987	81199.3		
Total	81029196.5	995			

**Figure 17:**
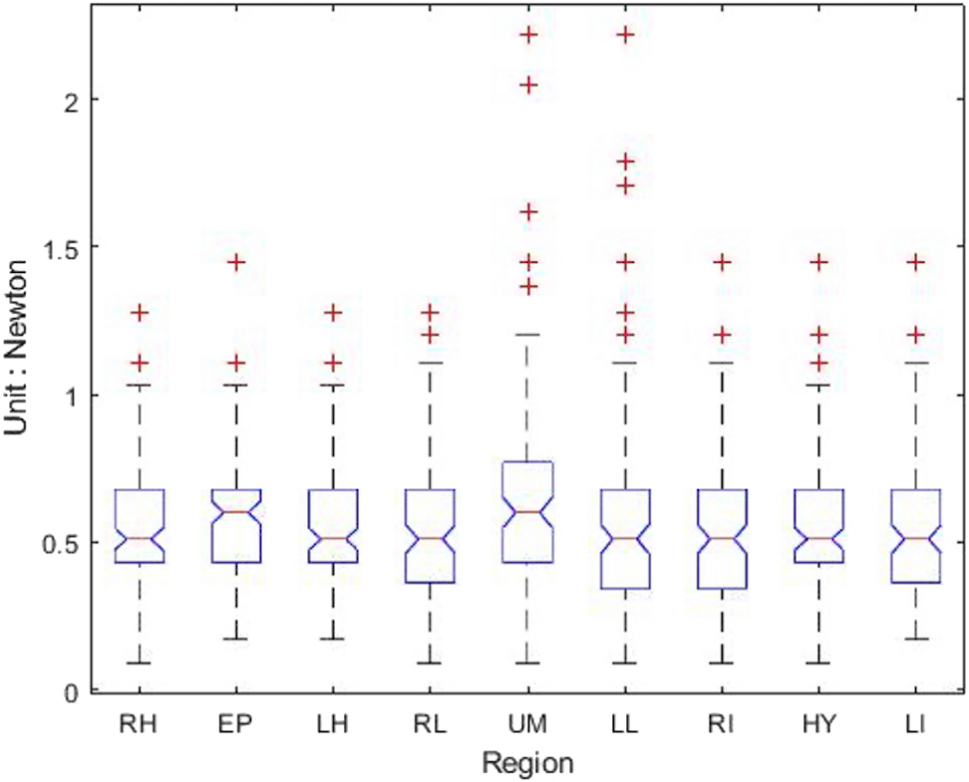
The box-plot of nine-region deep palpation for each group.

Similarly, Table 8 shows the output and Fig. 17 shows the box-plot of the Kruskal-Wallis test and whether there is a statistically significant difference between the nine groups of deep palpation. We can see that the *p*-value = 0.1135, which is above 0.05. Therefore, there is no statistically significant difference of deep palpation performed in each region.

We use points of anterior superior iliac spines and the cross of anterior axillary line and costal margin as anatomic landmark of abdomen, which may lose upper and lower part of the abdominal area. Adding xyphoid process and symphysis pubis as landmark points, which makes abdominal area as a hexagon area, may provide more precise information for clinical users.

Two episodes of contact dermal reaction were found, which may due to reaction with the material of the probe or the pressure sensor. Further attention for the contacting material may decrease the frequency of the episode.

The participants of our study were healthy people ranged between 23 and 49 years old. Further studies for children and elderly people may extend the application of our system.

Although there is a significant difference in mean pressure value between light and deep palpation, the maximal and minimal values in light palpation closed with the values of deep palpation. This may due to device factor, operation factor, or personal variation. Further evaluation is needed to improve the procedure.

With proper organization, our system has potential to become a tool providing digital information for abdominal palpation. It may also become a communication tool between patients and physicians in the telehealth era.

**Table 8:** The Kruskal-Wallis ANOVA Table of deep palpation in the nine-region scheme (variable: palpation pressure; groups: nine regions).

Source	SS	df	MS	Chi-sq	Prob>Chi-sq
Groups	1.07727e+06	8	134658.8	12.95	0.1135
Error	8.24271e+07	996	82758.2		
Total	8.35044e+07	1004			

## Conclusion

Walker et al. (1990) state that “Physical examination is the process of evaluating objective anatomic findings through the use of observation, palpation, percussion, and auscultation.” and “Palpation is the examination of the abdomen for crepitus of the abdominal wall, for any abdominal tenderness, or for abdominal masses.”

The purpose of the study is to measure applied force level of palpation pressure in the quantitative way rather than describe the force (such as light palpation and deep palpation) in the qualitative way. In this paper, we propose an inexpensive device of a pressure measurement system for abdominal physical examination to automatically locate positions, continuously keep track and quantify the pressure while performing palpations. With having a digitized mechanism, we might be able to unify the applied force level in more precise way. For example, during a palpation performed by the physician, if a provoking pain with a certain pressure is reported by patients, we are able to measure the pressure in the numerical format for better follow-up clinical study. Based on our clinical trials, we report the measurement of pressure values of light and deep palpation and indicate there is no significant difference between the four-quadrant and nine-region scheme. No significant difference among areas in the four-quadrant and nine-region scheme.

The limitation of the study is described as follows. In this study, we design a mechanism to quantify palpation force and keep track the position simultaneously. According to our experiment results shown in ‘Results’, we are able to conclude that a certain examiner is stable during the palpation process. However, without sufficient experiments with more examiners/physicians involved, we cannot establish the standard for palpation force level of deep and light palpation, respectively. Currently, in this study, we consider two palpation schemes (four quadrants and nine regions), but do not associate pressure measurements with the anatomic landmarks for the abdominal examinations. Moreover, when patients experience abdominal pain during the palpation process, it would be worth keeping track the pressure value at that moment. In addition, a localized or generalized unpleasant bodily sensation or complex of sensations that causes mild to severe physical discomfort and emotional distress and typically results from bodily disorder (such as injury or disease). Nevertheless, the complain of pain is of subjective sensation, informative, and patient-dependent in the examination. With having standardized and quantified measurements, it would be possible to achieve comparable studies of pain between patients. Whenever pain evoked by palpation, the different levels of palpation force should be assessed and standardized in numeric format. Once we have sufficient amount of experiment, the relationship between evoked pain and palpation force could be modeled for each individual. In the future research, we are considering to make use of IMU sensor (inertial measurement unit, IMU) to locate the sensing rod. Recently, the researchers have been employed IMU device for medical non-invasive diagnosis. For example, Glowinski et al. (2020) made use of inertial sensors to determine the pathologic parameters of gaits. In the current version of our system, we make use of camera to track the sensing rod to locate the palpation position. However, in some cases, the sensing rod might be blocked by the examiner (physician) such that the optical-based positioning mechanism fails. As a result, in the future study, we are considering to make use of IMU device, in cooperate with the camera, to provide a more reliable positioning mechanism.

##  Supplemental Information

10.7717/peerj.10511/supp-1 Supplemental Information 1 Raw data for deep palpations in the four quadrant scheme

10.7717/peerj.10511/supp-2 Supplemental Information 2 Raw data for light palpations in the four quadrant scheme

10.7717/peerj.10511/supp-3 Supplemental Information 3 Raw data for light palpations in the nine-region scheme

10.7717/peerj.10511/supp-4 Supplemental Information 4 Raw data for deep palpations in the nine-region scheme

10.7717/peerj.10511/supp-5 Supplemental Information 5 Program capturing the signal to estimate the position of sensing rod

10.7717/peerj.10511/supp-6 Supplemental Information 6 Video demonstration of our digital abdominal palpation pressure measurement and positioning device

## Additional Information and Declaration

### Data Availability

The following information was supplied regarding data availability: All experimental data and program code are available in the Supplemental Files.

## References

[ref-1] Ball JW, Dains JE, Flynn JA, Solomon BS, Stewart RW (2019). Seidel’s guide to physical examination: an interprofessional approach.

[ref-2] Castro MCF, Cliquet A (1997). A low cost instrumented glove for monitoring forces during object manipulation. IEEE Transactions on Rehabilitation Engineering.

[ref-3] Cha D, Kang D, Oh SN, Kim KI, Kim KS, Kim S (2013). Faster detection of step initiation for the unmanned technology research center exoskeleton (UTRCEXO) with insole-type force sensing resistor (FSR).

[ref-4] Farooq M, Sazonov E (2016). Linear regression models for chew count estimation from piezo- electric sensor signals. Proceedings of 10th International Conference on Sensing Technology (ICST).

[ref-5] Fenton BW, Palmieri PA, Durner C, Fanning J (2009). Quantification of abdominal wall pain using pain pressure threshold algometry in patients with chronic pelvic pain. The Clinical Journal of Pain.

[ref-6] FlexiForce A201 Sensor (2020). https://www.tekscan.com/products-solutions/force-sensors/a201.

[ref-7] Frantlović M, Smiljanić M, Lazić Ž (2016). Intelligent industrial measurement instruments with silicon piezoresistive MEMS pressure sensors.

[ref-8] Glowinski S, Łosiński K, Kowiański P, Waśkow M, Bryndal A, Grochulska A (2020). Inertial sensors as a tool for diagnosing discopathy lumbosacral pathologic gait: a preliminary research. Diagnostics.

[ref-9] Hollinger A, Wanderley M (2011). Evaluation of commercial force-sensing resistors. https://www.researchgate.net/publication/265922252_Evaluation_of_Commercial_Force-Sensing_Resistors.

[ref-10] Institute of Electrical and Electronics Engineers (1966). IEEE standard definitions and methods of measurement for piezoelectric vibrators. IEEE Std, 177.

[ref-11] Interlink Electronics (2020). FSR Model 408 (200mm length). https://buyinterlinkelectronics.com/collections/new-standard-force-sensors/products/fsr-model-408-200mm-length.

[ref-12] Jarvis C Physical examination and health assessment.

[ref-13] Kang AL, Wang WB, Liu YQ, Han T (2011). A wireless pressure sensor based on surface transverse wave.

[ref-14] Katsuki T, Youoku S, Mi X, Nakazawa F, Kawanabe T, Odaguchi H, Hanawa T (2015). A pressure/motion sensor system to quantify abdominal palpations in traditional medicine. Proceedings of 9th international conference on sensing technology (ICST).

[ref-15] Kinser AM, Sands WA, Stone MH (2009). Reliability and validity of a pressure algometer. The Journal of Strength & Conditioning Research.

[ref-16] Ko SJ, Kim H, Kim SK, Park K, Lee J, Lee BJ, Oh J, Lee K, Park JW (2016). Reliability and validity of modified algometer in abdominal examination. Evidence-Based Complementary and Alternative Medicine.

[ref-17] Krklješ D, Nagy L, Babkovič K (2012). Force-dependent contact area excitation of FSR force sensor utilizing dome-shaped rubber element.

[ref-18] Ling TW (1999). A study on signal processing circuits of piezoelectric pressure sensors. Master’s thesis.

[ref-19] Masuda M, Iida T, Exposto FG, Baad-Hansen L, Kawara M, Komiyama O, Svensson P (2018). Referred pain and sensations evoked by standardized palpation of the masseter muscle in healthy participants. Journal of Oral & Facial Pain and Headache.

[ref-20] Myers DR, Weiss A, Rollins MR, Lam WA (2017). Towards remote assessment and screening of acute abdominal pain using only a smartphone with native accelerometers. Scientific Reports.

[ref-21] Nam H, Kim JH, Kim JI (2016). Smart belt: a wearable device for managing abdominal obesity.

[ref-22] Niederauer S, Cottle B, Sheng X, Ashton-Miller J, Delancey J, Hitchcock R (2020). Subsequent use of a pressure sensor to record intra-abdominal pressure after maximum vaginal closure force in a clinical trial. IEEE Journal of Translational Engineering in Health and Medicine.

[ref-23] Ogris G, Kreil M, Lukowicz P (2007). Using FSR based muscule activity monitoring to recognize manipulative arm gestures.

[ref-24] Pacchierotti C, Prattichizzo D, Kuchenbecker KJ (2015). Cutaneous feedback of fingertip deformation and vibration for palpation in robotic surgery. IEEE Transactions on Biomedical Engineering.

[ref-25] Park J, Kim SJ, Na Y, Kim J (2016). Custom optoelectronic force sensor based ground reaction force (GRF) measurement system for providing absolute force.

[ref-26] Praveen K, Guhilot H (2013). FPGA implementation of contactless position sensor using FSR (Force sensing Resistor).

[ref-27] Research and Markets (2019). Global force sensor market analysis & trends 2015–2017 & industry forecasts 2017–2027. https://www.businesswire.com/news/home/20190403005484/en/Global-Force-Sensor-Market-Analysis-Trends-2015-2017-Industry-Forecasts-2017-2027---ResearchAndMarkets.com.

[ref-28] Sepúlveda AT, Pontes AJ, Viana JC, GuzmandeVilloria R, Fachin F, Wardle BL, Rocha LA (2011). Flexible sensor for blood pressure measurement.

[ref-29] Shaby SM, Juliet AV (2011). Performance analysis and validation of sensitivity of piezoresistive MEMS pressure sensor.

[ref-30] Sonkar SK, Suja K, Krishnan JS (2014). Signal conditioning unit for piezoresistive based micro pressure sensors.

[ref-31] Swartz MH (2002). Textbook of physical diagnosis: history and examination.

[ref-32] Tokuyasu T, Nakayama T, Toshimitsu K, Okamura K, Yoshiura K Development of virtual palpation system for dental education.

[ref-33] Vecchi F, Freschi C, Micera S, Sabatini A, Dario P, Sacchetti R (2001). Experimental evaluation of two commercial force sensors for applications in biomechanics and motor control.

[ref-34] Walker HK, Hall WD, Hurst JW (1990). Clinical methods: the history, physical, and laboratory examinations.

[ref-35] Więckiewicz W, Woźniak K, Piątkowska D, Szyszka-Sommerfeld L, Lipski M (2015). The diagnostic value of pressure algometry for temporomandibular disorders. BioMed Research International.

[ref-36] Wytrązek M, Huber J, Lipiec J, Kulczyk A (2015). Evaluation of palpation, pressure algometry and electromyography for monitoring trigger points in young participants. Journal of Manipulative and Physiological Therapeutics.

[ref-37] Yoshitomi K, Tokuyasu T, Toshimitsu K, Nakayama T, Okamura K, Yoshiura K (2016). Improvement of mesh free deforming analysis for maxillofacial palpation on a virtual training system.

[ref-38] Yu PJ (2014). Physical examination and assessment.

[ref-39] Zhang Y, Feng D, Liu Z, Guo Z, Dong X, Chiang KS, Chu BCB (2001). High-sensitivity pressure sensor using a shielded polymer-coated fiber Bragg grating. IEEE Photonics Technology Letters.

